# Normobaric oxygen paradox and erythropoietin production in critically ill patients: a prospective observational study

**DOI:** 10.1186/cc14400

**Published:** 2015-03-16

**Authors:** S Zuccari, A Donati, E Damiani, R Castagnani, N Mininno, P Pelaia

**Affiliations:** 1 Università Politecnica delle Marche, Ancona, Italy

## Introduction

The normobaric oxygen paradox (NOP) postulates that a period of normobaric hyperoxia followed by a rapid return to normoxia will create a condition of relative hypoxia, which acts in turn as a stimulus for erythropoietin (EPO) production [1]. Variations in GSH and oxygen free radical (ROS) levels may be involved in this process. We tested the NOP in critically ill patients.

## Methods

A prospective observational study on 38 mechanically ventilated (FiO_2_ <50%) patients with no active bleeding, no blood transfusion needed, and no kidney failure. Eighteen patients underwent a 2-hour period of normobaric hyperoxia (FiO_2 _= 100%), 20 patients were evaluated as controls (no FiO_2_ variations). EPO was assayed at baseline (t0), 24 hours (D1) and 48 hours (D2). Serum GSH and ROS were assayed at t0 (baseline), t1 (2-hour FiO_2_ 100%) and t2 (2hour return to normoxia) in 12 patients in the hyperoxia group.

## Results

**Figure 1:**
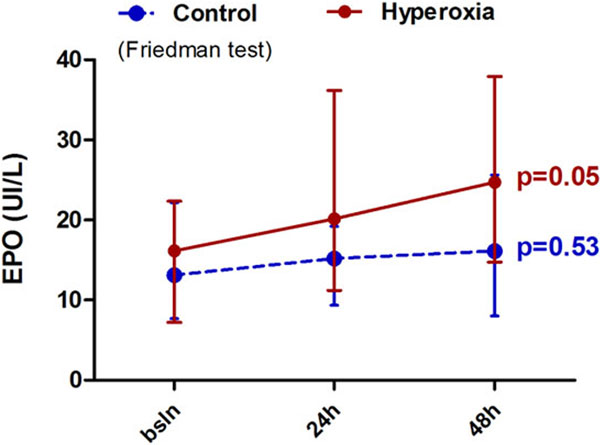


EPO tended to increase in the hyperoxia group over time (*P *= 0.05), while it remained stable in the control group (*P *= 0.53) (Figure 1). ROS levels increased at t1 and decreased at t2, GSH tended to decrease at t1 and increased at t2 in the hyperoxia group.

## Conclusion

Relative hypoxia after a transient period of normobaric hyperoxia induces an increase in GSH levels, thus enhancing ROS scavenging. This may act as a stimulus for EPO production.

## References

[B1] Balestra J Appl Physiol 2006 100 512 8 10.1152/japplphysiol.00964.2005 16239610

